# Supplementary material for: Local maximum synchrosqueezes form scaling-basis chirplet transform
Source: PLoS One. 2022 Nov 29;17(11):e0278223. doi: 10.1371/journal.pone.0278223 (PMC9707797; doi:10.1371/journal.pone.0278223)
Supplement: Attachment — Submitted filename: Response to Reviewers.docx [file pone.0278223.s001.docx]

**Paper Title:** Local maximum synchrosqueezes form scaling-basis chirplet transform

**Authors:** Yating Hou, Liming Wang, Xiuli Luo, Xingcheng Han

**Manuscript Number:** PONE-D-22-22608

**Date:** November 7, 2022

**Response to the Reviewers**

We would like to thank the reviewers and the editor for their time in reviewing our paper and suggestions for improving it. In this document, we have written a detailed point-by-point response to the reviewers to address their comments and explain the corresponding changes made in the manuscript.

The detailed point-by-point responses to journal requirements are given as follows:

(1) We amend our manuscript to meet PLOS ONE's style requirements according to the PLOS ONE style templates;

(2) In financial disclosure, the funders assisted with study design and manuscript preparation;

(3) We amend our Data Availability Statement and provide the corresponding URLs where the datasets can be accessed;

The Bat signal used in this paper came from Rice University. The website is https://web.archive.org/web/20160403234536/http://dsp.rice.edu/software/bat-echolocation-chirp.

The Vibration signals from Case Western Reserve Univesity dataset. The website is https://engineering.case.edu/bearingdatacenter/apparatus-and-procedures.

(4) We have created an ORCID iD and updated my Information;

(5) We were sorry for our careless mistakes. We ensure that all figures were mentioned in the manuscript.

The detailed point-by-point responses to each reviewer’s comments are given in the rest of this document. Throughout this document, we use the following conventions:

- *Blue text in Italics* = Reviewers’ comments.
- Red text = New additions to the manuscript.
- Black text = Authors’ responses.

This response letter is a self-contained document that conveniently includes all the revisions.

We hope that the current revision meets your approval.

Kind regards,

Yating Hou, Liming Wang, Xiuli Luo, Xingcheng Han

We would like to thank the reviewers for their valuable comments, which have helped us to further improve the contribution of the paper. We have addressed all the comments and suggestions and revised our manuscript as follows:

**For Reviewer: 1**

**Response to the comments**

**Comment 1:** *In order to better compare the TF concentration of the TFR obtained different TF methods, it is suggested to display the RE values of each component in the table as EMD.*

**Our Response:** The authors sincerely appreciate the valuable comments. EMD is to divide the nonstationary signal into several intrinsic mode functions from high to low frequencies, and then perform Hilbert transform on each IMF component of the decomposition. The method used in this study is to choose a short window for the signal to be observed. It is assumed that the signal within each short window is stationary, but each window may contain more than one prominent frequency component. It is difficult to evaluate Rényi entropy for each component. In order to better evaluate the processing performance of different methods for each frequency component, this paper shows the instantaneous amplitude spectra of several methods at the time of 0.5s.

**Actions on Manuscript:**

In Section 4.2, the added figure 6 is shown below [p17, line293]:

|  |  |
| --- | --- |
| **(a)** | **(b)** |
|  |  |
| **(c)** | **(d)** |
| **Fig 6. TF slices obtained through (a) STFT, (b) SBCT, (c) RM, and (d) LMSBCT at time t=0.5 s.** | |

In the third paragraph of Section 4.2, the following sentences have been added to explain Fig 6 and improve the quality of the paper [p16, line285-292]:

Fig 6 illustrates the instantaneous amplitude spectra of different time-frequency analysis methods at the time of 0.5 s. The spectral bandwidths of STFT and SBCT are large, and there is no boundary between the frequency components. In contrast, the energy of the frequency spectrum of RM and LMSBCT is concentrated in a narrow bandwidth. The frequency component IF3 was locally amplified (represented in red). Comparing Figs 6(c) and (d), LMSBCT has clear boundaries between each frequency component, more concentrated energy, and high noise immunity.

**Comment 2:** *The authors should introduce more details of the experimental device and the experimental implementation, rather than just give the data source.*

**Our Response:** The authors sincerely appreciate the valuable comments. The authors have checked the literature carefully and added more details about the experimental device and the experimental implementation in section 5 of the revised manuscript.

**Actions on Manuscript:**

In Section 5.1, “Digitized echolocation pulse emitted by the Large Brown Bat, *Eptesicus Fuscus.*” was added [p20, line339-340].

To better show the effectiveness of the algorithm in this paper, the authors changed this part of the processed signal to a multicomponent bat echo signal. Due to the newly added pictures before, figure 9 and figure 10 become figure 10 and figure 11 in order. Figures 10 and 11[p21, line355-356] were replaced with the following figures. Table 3[p22, line357] was replaced with the following Table 3.

|  |  |
| --- | --- |
| **(a)** | **(b)** |
|  |  |
| **(c)** | **(d)** |
| **Fig 10. TFA results obtained by(a) STFT, (b) GLCT, (c) SBCT, and (d)SET** | |

**Fig 11.TFA result obtained by LMSBCT.**

**Table 3. Rényi Entropy of different algorithms**

| Method | | STFT | SBCT | GLCT | SET | LMSBCT |
| --- | --- | --- | --- | --- | --- | --- |
| Rényi entropy | 13.9721 | | 11.0362 | 15.3418 | 9.8795 | 8.3940 |

In Section 5.2, The authors added the experimental setup diagram [p24, line383-384] and the data acquisition method [p23, line362-367]. The following figure shows the experimental setup diagram. Then the installation location of the device and the data acquisition method are described separately.

**Fig 12. CWRU teststand.**

Experiments were carried out using a 2-horsepower Reliance Electric motor with acceleration data measured near and away from the motor bearings. As shown in Fig 12, the test stand included a 2 hp motor, torque transducer/encoder, dynamometer, and control electronics. Vibration data were collected using accelerometers attached to housing with magnetic bases.

**Comment 3:** *In Section 6, it is suggested that the authors also use quantitative indicators to measure the TFR results of different TF analysis methods and analyze them.*

**Our Response:** The authors sincerely appreciate the valuable comments. The authors have rewritten this part according to the Reviewer’s suggestion.

**Actions on Manuscript:** The authors again use the Rényi entropy indicators to highlight the superiority of the proposed algorithm. This action will make the conclusions more objective and credible. The revised paragraph[p25, line396-403] reads as follows：

(1) The frequency changes of strong time-varying signals can be analyzed effectively. Compared with other TFA methods, the Rényi entropy of LMSBCT can be reduced to 9.6438. (2) The Rényi entropies of the LMSBCT algorithm were always lower than those of the other methods when the SNR was reduced from 30 dB to 1 dB. This implies that the multicomponent signals can be effectively separated, even at low SNRs. (3) This method can also obtain an elaborate TFR when the instantaneous frequencies of the signals are close to each other. Even if the frequency interval of the signal is less than 1Hz, the Rényi entropy of the LMSBCT is the smallest compared to the other methods, which is 8.0927.

**For Reviewer: 2**

**Response to the comments**

**Comment 1:** *The authors should state the contribution and the novelty of this paper. The proposed method LMSBCT is a simple combination of SBCT and LMSST.*

**Our Response:** The authors sincerely appreciate the valuable comments. LMSST is essentially a post-processing method of STFT. Fundamentally, the effectiveness of these post-processing methods and the superiority of the processed signals also depend on the original time-frequency analysis method. SBCT is also an improved method of STFT. Our manuscript proposes the LMSBCT method by making a good combination of SBCT and LMSST for the first time based on the improvement of STFT, i.e., SBCT. Such an approach would give the results of time-frequency analysis the advantages of SBCT as well as the advantages of increased post-processing. Simulated and real-life signals are employed to validate the advantages of LMSBCT by comparing them with some advanced TF methods. The experimental results demonstrate that LMSBCT can provide a better time-varying description, obtain a more precise IF estimate, and have stronger noise robustness.

**Actions on Manuscript:** The authors have added a sentence to the Abstract section to clarify the novelty of this paper. The added sentence is shown below [p1, line17-18]:

which is a further improvement of the scaling-basis chirplet transform (SBCT) with energy rearrangement in frequency and can be viewed as a good combination of SBCT and local maximum synchrosqueezing transform.

**Comment 2:** *The English level of this paper should be improved.*

**Our Response:** The authors sincerely appreciate the valuable comments. The authors revised and improved the English of our manuscript. The authors have polished our article by editage, as shown below.

Thanks again for your thoughtful suggestion, it is very important. Due to your suggestion, we found some shortcomings in our current work. We will improve our research level according to your suggestion in future work and get more achievements. We are hoping to learn more from you.
